# Uncontrolled and apparent treatment resistant hypertension: a cross-sectional study of Russian and Norwegian 40–69 year olds

**DOI:** 10.1186/s12872-020-01407-2

**Published:** 2020-03-13

**Authors:** Jakob Petersen, Sofia Malyutina, Andrey Ryabikov, Anna Kontsevaya, Alexander V. Kudryavtsev, Anne Elise Eggen, Martin McKee, Sarah Cook, Laila A. Hopstock, Henrik Schirmer, David A. Leon

**Affiliations:** 1 London School of Hygiene & Tropical Medicine, WC1E 7HT, London, UK; 2 Research Institute of Internal and Preventive Medicine, Branch of Institute of Cytology and Genetics, Siberian Branch of the Russian Academy of Sciences, Novosibirsk, 630090 Russia; 3 Novosibirsk State Medical University, Russian Ministry of Health, Novosibirsk, 630091 Russia; 4 National Research Center for Preventive Medicine, Ministry of Healthcare, Moscow, Russia; 5 Northern State Medical University, Arkhangelsk, 163000 Russia; 6 Department of Community Medicine, UiT The Arctic University of Norway, 9037 Tromsø, Norway; 7 University of Oslo, Institute for clinical medicine, 1171 Blindern, 0318 Oslo, Norway; 8 Department of Cardiology, Akershus University Hospital, 1478 Nordbyhagen, Oslo, Norway; 9 International Laboratory for Population and Health, National Research University, Higher School of Economics, Moscow, Russia

**Keywords:** Cardiovascular diseases, Hypertension, Antihypertensive agents

## Abstract

**Background:**

Uncontrolled hypertension is a major cardiovascular risk factor. We examined uncontrolled hypertension and differences in treatment regimens between a high-risk country, Russia, and low-risk Norway to gain better understanding of the underlying factors.

**Methods:**

Population-based survey data on 40–69 year olds with hypertension defined as taking antihypertensives and/or having high blood pressure (140+/90+ mmHg) were obtained from Know Your Heart Study (KYH, *N* = 2284), Russian Federation (2015–2018) and seventh wave of The Tromsø Study (Tromsø 7, *N* = 5939), Norway (2015–2016). Uncontrolled hypertension was studied in the subset taking antihypertensives (KYH: *N* = 1584; Tromsø 7: 2792)and defined as having high blood pressure (140+/90+ mmHg). Apparent treatment resistant hypertension (aTRH) was defined as individuals with uncontrolled hypertension on 3+ OR controlled on 4+ antihypertensive classes in the same subset.

**Results:**

Among all those with hypertension regardless of treatment status, control of blood pressure was achieved in 22% of men (KYH and Tromsø 7), while among women it was 33% in Tromsø 7 and 43% in KYH. When the analysis was limited to those on treatment for hypertension, the percentage uncontrolled was higher in KYH (47.8%, CI 95 44.6–50.9%) than Tromsø 7 (38.2, 36.1–40.5%). The corresponding figures for aTRH were 9.8% (8.2–11.7%) and 5.7% (4.8–6.8%).

Antihypertensive monotherapies were more common than combinations and used by 58% in Tromsø 7 and 44% in KYH. In both KYH and Tromsø 7, untreated hypertension was higher in men, those with no GP visit in the past year and problem drinkers. In both studies, aTRH was associated with older age, CVD history, obesity, and diabetes. In Tromsø 7, also male gender and any drinking. In KYH, also chronic kidney disease.

**Conclusion:**

There is considerable scope for promoting combination therapies in line with European treatment guidelines in both study populations. The factors associated with untreated hypertension overlap with known correlates of treatment non-adherence and health check non-attendance. In contrast, aTRH was characterised by obesity and underlying comorbidities potentially complicating treatment.

## Background

Mortality from cardiovascular disease (CVD) has been falling rapidly in Russia since 2005 [1]. This is thought to be due, in part, to better detection, treatment, and control of hypertension [2–4]. Yet, despite this impressive progress, control of blood pressure remains relatively poor and there is a persisting mortality gap with countries of Western Europe. At first sight, there is no good reason for this. Russia has an extensive health system with, in comparative terms, large numbers of health workers [5]. Hypertension is easily diagnosed and can be treated with a range of safe and effective medicines [6], widely available in Russian pharmacies [7].

An effective response is clearly needed but this must be informed by a detailed understanding of why Russia has been unable to achieve better control of blood pressure. This study seeks to inform such a response by examining in detail the characteristics of a population sample of Russians, who have been initiated on antihypertensive treatment but whose blood pressure remains uncontrolled. It compares them with similarly defined individuals from neighbouring Norway to assess whether there are lessons that can be learnt from its comparatively better control of blood pressure.

In the study we compare uncontrolled and apparent treatment resistant hypertension among those taking antihypertensives in population-based samples from Russia and Norway as well as the proportion of individuals achieving blood pressure control among all with hypertension.

## Methods

### Data selection criteria

Know Your Heart (KYH) is a cross-sectional, population-based study of cardiovascular structure, function, and risk factors in over 4500 men and women aged 35–69 years living in two Russian cities, Arkhangelsk and Novosibirsk, 2015–2018 [8]. The Tromsø Study is a longitudinal, population-based, prospective study with repeated data collections since 1974 in the municipality of Tromsø in Northern Norway. Data from the seventh wave of the study, Tromsø 7 (2015–2016), were used [9].

A total of 2284 and 5939 participants were selected for this study from KYH and Tromsø 7, respectively, based on the following inclusion criteria: aged 40–69 years, non-missing systolic and diastolic blood pressure measurement, measured hypertension (systolic pressure of 140+ mmHg and/or a diastolic of 90+ mmHg) or taking antihypertensives [6]. A subset of participants, those taking antihypertensives (1584 from KYH and 2792 from Tromsø 7), were then selected to study uncontrolled and apparent treatment resistant hypertension. A flow diagram of the sample selection is presented in Figure S1 (Supplementary materials). Response rates for the health check component in KYH was 67% in Arkhangelsk and 37% in Novosibirsk [8] (denominator: all issued, excluding addresses not found or where no one of target age and gender were found). The response rate in Tromsø 7 was 65% [9].

### Blood pressure measurement

In KYH, blood pressure was measured using OMRON 705 IT blood pressure monitors (OMRON Healthcare, Kyoto, Japan). All devices were calibrated before and after the fieldwork period and no adjustments were needed. Blood pressure in the Tromsø 7 study was measured using Dinamap ProCare 300 blood pressure monitors (GE Healthcare, Oslo, Norway) calibrated before the fieldwork. In both studies three measurements were taken with two minutes seated rest in between [8]. Participants were assigned to different antihypertensive classes based on their systolic and diastolic blood pressure (average of last two out of three measurements) according to the European hypertension treatment guidelines [6] and antihypertensive use.

### Medication

Participants in both Know Your Heart (KYH, Russian Federation) and Tromsø 7 (Norway) were asked questions about their use of antihypertensives, although the protocols differed in some minor respects. In KYH, a baseline interview was administered by a trained non-medical interviewer. Participants who reported ever being diagnosed with hypertension were asked a series of questions about prescription and use of blood pressure medication. At the end of the interview all participants were invited to attend a health check to which they were asked to bring all their medications. At the health check, a trained medical interviewer asked the participant about current medication use and recorded the name, dose, indication and frequency of use of medications (up to 7 medications). About a third (33%) of KYH participants brought their medicines with them to the health assessment and for the remaining two thirds (67%), the record taking was verbatim. In Tromsø 7, all participants were asked about current or previous use of antihypertensive medications. Furthermore, participants were asked to state the name of all medications (prescription and non-prescription drugs) they had used regularly during the last four weeks (up to 20 medications). The questionnaire was checked by a trained technician at the study site, and participants had to confirm if no medication use was reported. For both studies listed medications were coded using the International WHO Anatomical Therapeutic Chemical (ATC) classification system [10]. Antihypertensive medication was defined as medications within the following ATC classes: Antihypertensives (abbreviation: AH; ATC code: C02), Diuretics (DIU; C03), Beta blockers (BB; C07), Calcium channel blockers (CCB; C08), ACE inhibitors (ACE; C09A/B), Angiotensin II receptor blockers (ARB; C09C/D).

The main analyses were based on ATC coding of reported medications. Sensitivity analyses were conducted using self-reported positive response to an explicit question about whether the participant regularly took antihypertensive medication.

### Outcome

Uncontrolled hypertension was studied in those on antihypertensive medication in two different ways. First, as uncontrolled hypertension in the usual sense, i.e. individuals on antihypertensives and with systolic blood pressure 140+ mmHg and/or a diastolic of 90+ mmHg. Second, as apparent treatment resistant hypertension (aTRH), i.e. individuals with uncontrolled hypertension on 3+ OR controlled on 4+ antihypertensive classes [11]. In a clinical setting, a patient would only be diagnosed with treatment resistant hypertension following a medication review, an assessment of medication adherence, and a series of elevated out-of-office blood pressure measurements. All of these factors are rarely present in large epidemiological studies and aTRH has shown to provide useful insights in various studies including NHANES studies [11, 12].

The prevalence of uncontrolled hypertension and aTRH among those with treated hypertension was estimated and standardised by age and sex to the 2013 European Standard Population [13].

### Co-variates

A range of co-variates was included to study factors associated with hypertension, CVD risk, CVD prevention, and healthcare system use, i.e. gender, age, education, whether living with a partner, body mass index, alcohol use, CVD history, and primary healthcare visits. The equivalent co-variates from KYH and Tromsø 7 were harmonised in terms of coding frames and standard classifications.

Chronic kidney disease (CKD) status was defined as Glomerular Filtration Rate (eGFR) below 60 ml/min/1.73m^2^ based on serum creatinine [14].

Self-reported alcohol-related behaviours were categorised as: non-drinker past year versus low risk drinkers (score < 8) versus high risk drinkers (score 8+) according to WHO Alcohol Use Disorders Identification Test (AUDIT) [15].

For KYH, the responses to a question on household financial constraints were classed into the following categories: perceived to be constrained in buying food or clothes, able to buy food or clothes but constrained in buying large domestic appliances, able to buy both. No equivalent variable was available from Tromsø 7.

The presence of diabetes was ascertained on either self-reported diabetes or taking diabetes medication (ATC A10: insulin or oral antidiabetics) or HbA1c 48+ mmol/mol (> 6.5%) [16].

Serum total cholesterol (mmol/L) was combined with data on age, sex, smoking status, and systolic blood pressure to calculate 10-year risk of a fatal CVD event according to the SCORE tool equations for high risk countries and divided into three risk groups for the descriptive analyses: Low (< 1%), Moderate (1–4.9%), High (5 + %) [17] (pers.comm. Dr. T. Fitzgerald for additional information regarding the Conroy et al. (2003) risk equations in line with the European Cardiology Society’s online CVD risk calculator).

A history of CVD was defined as self-report of one or more of the following conditions: myocardial infarction, heart failure, atrial fibrillation, angina, stroke.

Whether the participant had visited a primary care consultant in the past year was also included (KYH: general practice or polyclinic; Tromsø 7: general practice).

Biomarker data from KYH (total cholesterol, HbA1c, serum creatinine) were corrected for inter-laboratory variation (Iakunchykova O, Averina M, Wilsgaard T, Leon DA: Recalibration of Blood Analytes in Know Your Heart Study for Comparisons with Tromsø 7 study - Impact of Recalibration on Mean Levels and Prevalence estimates, in preparation).

### Regression analyses

Multivariate logistic regression models of uncontrolled versus controlled hypertension and aTRH versus non-aTRH in 40–69 year olds with treated hypertension were fitted adjusting for gender and age, or gender, age, and CVD history while including the following covariates: study, education, body mass index, smoking, alcohol consumption, diabetes, CKD, and primary healthcare visits. Statistical analyses were carried out using Stata 15 [18]. CVD history was included in the adjusted analyses together with gender and age due to the well-known differences in CVD burden between the two countries and the higher propensity for being and staying on antihypertensives for this group [19]. Potential interactions between age group, gender, age group, and CVD history for the two outcomes were tested with likelihood ratio tests in a restricted versus unrestricted model scenario.

## Results

A total of 1584 and 2792 40–69 year olds were on antihypertensives in KYH and Tromsø 7, respectively (for the characteristics of study participants, see Table 1). Individuals in Russia were more likely to have uncontrolled hypertension, at 55.7% (95% CI 50.6–60.6) in males and 42.7% (38.9–46.7) in females than in Norway (Table 2), where the corresponding percentages were 43.6% (40.4–46.8) and 33.0% (30.2–35.9). The proportion of people with hypertension who had aTRH was also higher in Russia, at 10.8% (8.1–14.1) in males and 9.2% (7.3–11.6) in females (Table 2). The corresponding shares in Tromsø 7 were 6.9% (5.4–8.7) and 4.5% (3.4–5.9). Controlling for age, gender, and CVD history, these gaps persisted, with adjusted odds ratios (AOR: KYH/Tromsø 7) of 1.65 (95% CI 1.45–1.87) for uncontrolled hypertension and 1.58 (1.26–1.98) for aTRH (Table 2). The gaps also persisted when controlling for a wider range of co-variates (Table 2). The sensitivity analyses based on participants’ response to a question about taking antihypertensives rather than the ATC-coding of self-reported medication used in the main analyses yielded similar results (Table 2).

**Table 1:** Characteristics of participants by study (age- and sex-standardised to 2013 European Standard Population)

Characteristic	Level	KYH		Tromsø 7	
		N	%	N	%
Study total	Total	1584	100	2792	100
Age group	40–44 yr	80	5.1	151	5.4
	45–49 yr	125	7.9	273	9.8
	50–54 yr	198	12.5	334	12.0
	55–59 yr	296	18.7	502	18.0
	60–64 yr	405	25.6	720	25.8
	65–69 yr	480	30.3	812	29.1
Gender	Male	598	38.0	1454	50.8
	Female	986	62.0	1338	49.2
Education	Elementary	245	12.9	744	21.7
	Lower intermediate	204	12.1	891	33.5
	Higher intermediate	652	42.4	524	20.5
	Graduate	483	32.5	611	24.4
Economic activity	Paid work	248	34.5	1668	71.5
	Looking after home	152	7.1	16	0.4
	Unemployed	24	2.8	15	0.7
	Retired	1145	53.9	551	10.0
	Other	15	1.6	504	17.3
Financial constraints	Constrained	365	22.0	N/A	N/A
	Intermediary	800	50.2	N/A	N/A
	Rel. unconstrained	398	27.8	N/A	N/A
Single	No	1039	67.4	2064	77.8
	Yes	545	32.6	595	22.2
Smoking	No	1262	74.4	2394	87.0
	Yes	320	25.6	356	13.0
Alcohol use disorder	Non-drinker past year	1145	67.6	1398	52.3
	Low (AUDIT< 8)	284	20.8	865	33.9
	High (AUDIT 8+)	150	11.5	322	13.8
Body Mass Index	Under/Normal (< 25)	226	15.7	498	17.3
	Overweight (25–29)	572	34.3	1167	39.0
	Obese (30–34)	489	29.8	765	28.5
	Very obese (35+)	293	20.1	344	15.2
Diabetic	No	1182	79.7	2366	84.9
	Yes	402	20.3	426	15.1
SCORE 10-year CVD risk%	Low (< 1%)	232	33.8	466	34.7
	Intermediate (1–4.9%)	755	44.5	1325	45.6
	High (5 + %)	564	21.7	949	19.7
Seen GP past year	No	301	19.1	192	7.1
	Yes	1283	80.9	2589	92.9
CKD	No	1516	96.7	2694	97.2
	Yes	68	3.3	98	2.8
CVD history	No	910	65.9	2100	79.5
	Yes	674	34.1	692	20.5

**Table 2:** Prevalence of uncontrolled and an apparent treatment resistant hypertension (aTRH) and odds ratios (AOR; 95% CI) for inter-study effects

Class		KYH (Russia)	Tromsø 7 (Norway)	KYH:Tromsø 7 AOR	KYH:Tromsø 7 AOR	KYH:Tromsø 7 AOR
				Age/Sex	Age/Sex/CVD	Multiple^b^
Uncontrolled hypertension	Male	55.7 (50.6 to 60.6)	43.6 (40.4 to 46.8)	1.90 (1.56;2.30)	2.03 (1.67;2.47)	1.97 (1.59;2.44)
	Female	42.7 (38.9 to 46.7)	33.0 (30.2 to 35.9)	1.30 (1.10;1.54)	1.35 (1.14;1.61)	1.22 (1.01;1.49)
	Total	47.8 (44.6 to 50.9)	38.2 (36.1 to 40.5)	1.54 (1.36;1.75)	1.65 (1.45;1.87)	1.59 (1.39;1.83)
	Total^a^	48.7 (45.2 to 52.2)	41.7 (39.5 to 44.0)	N/A	N/A	N/A
aTRH	Male	10.8 (8.1 to 14.1)	6.9 (5.4 to 8.7)	1.62 (1.18;2.21)	1.50 (1.09;2.06)	1.58 (1.12;2.24)
	Female	9.2 (7.3 to 11.6)	4.5 (3.4 to 5.9)	2.05 (1.50;2.80)	1.61 (1.16;2.23)	1.43 (0.98;2.08)
	Total	9.8 (8.2 to 11.7)	5.7 (4.8 to 6.8)	1.83 (1.47;2.28)	1.58 (1.26;1.98)	1.49 (1.16;1.91)
	Total^a^	10.2 (8.4 to 12.3)	5.5 (4.5 to 6.6)	N/A	N/A	N/A

^a^) Sensitivity analysis based on self-reported antihypertensive use where the main analyses were based on ATC coding of participant medication. ^b^) Age, sex, CVD history, smoking, AUDIT, BMI, Diabetes, Seen GP past year, CKD

For KYH, the proportions with controlled hypertension (defined as blood pressures < 140/< 90 mmHg and taking antihypertensives) out of all those with hypertension were 22.2% (19.6–25.1) for males and 43.0% (39.7–46.3) for females. For Tromsø 7, the equivalent figures were similar for males at 22.4% (21.0–23.8), but lower for females, 32.6% (30.6–34.8).

**Fig. 1:**
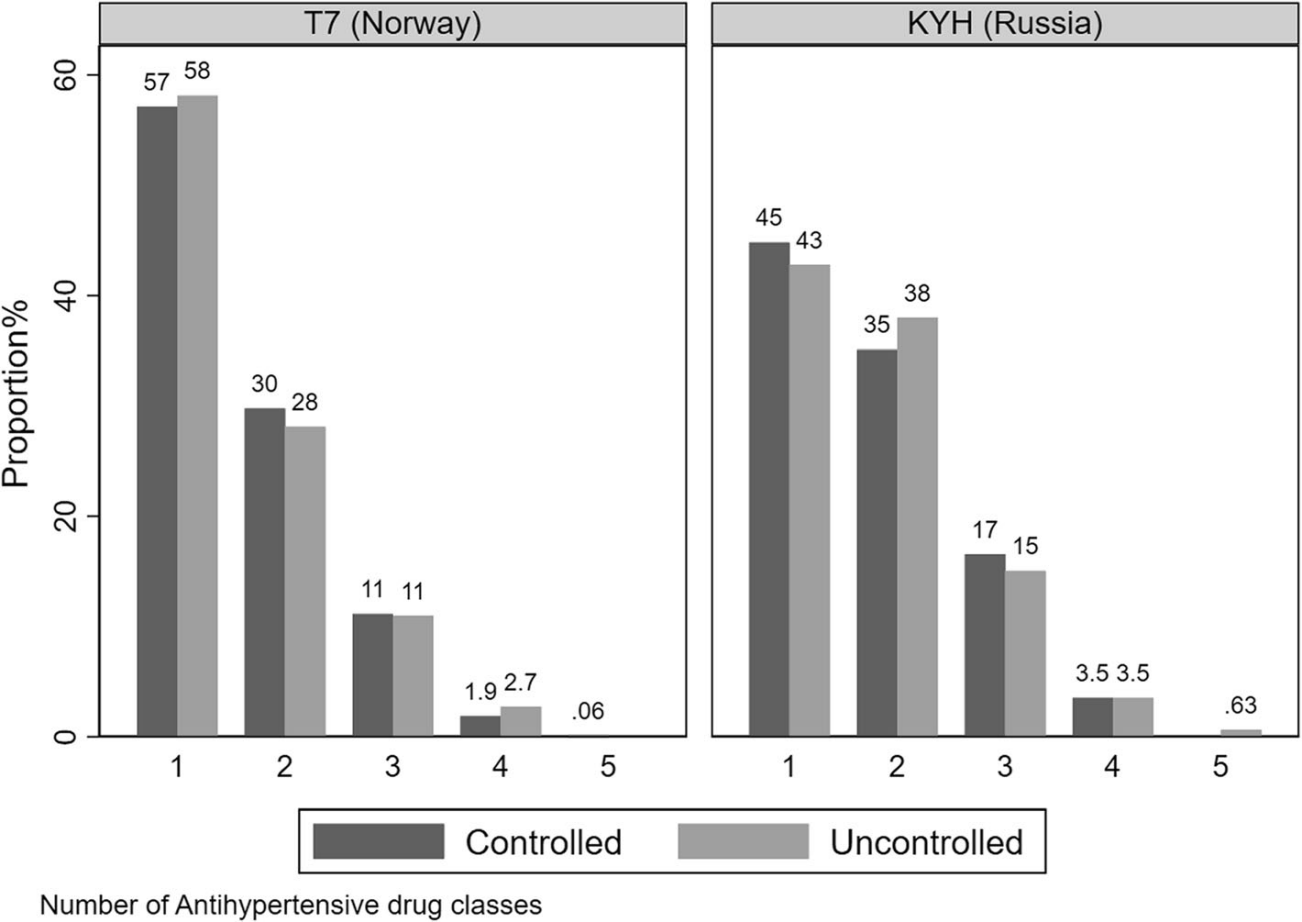
Number of antihypertensive drug classes used by study and whether hypertension was controlled or not

The frequency of antihypertensive use by drug class and whether hypertension was controlled or not showed that monotherapies were used in Tromsø 7 by as many as 58% of participants, whereas in KYH the same proportion was only 44%. While there was a declining number of participants for each additional drug class used, KYH had a longer right-hand tail, i.e. synonymous with greater use of multiple therapies than in Tromsø 7 (Fig. 1).

**Fig. 2:**
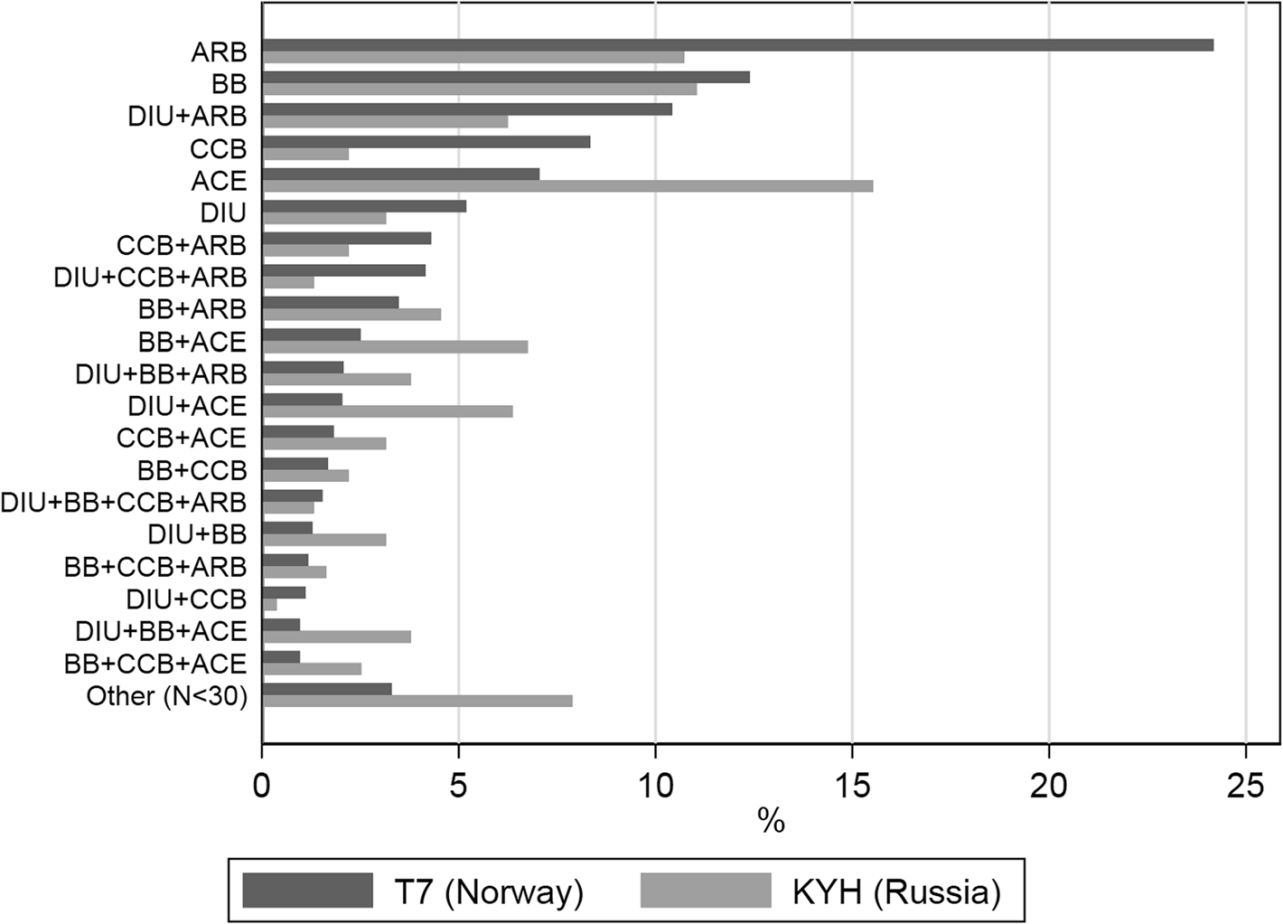
Antihypertensive use by drug class and study. AH = Antihypertensives (ATC code: C02), DIU = Diuretics (C03), BB=Beta blockers (C07), CCB=Calcium channel blockers (C08), ACE = ACE inhibitors (C09A/B), ARB = Angiotensin II receptor blockers *(C09C/D)*

ACE inhibitors, ARB, and BB were widely used as monotherapies in both studies; ARB (24.2% of all with treated hypertension) was the most commonly used therapy in Tromsø 7, while ACE (15.5%) was the most common in KYH (Fig. 2). In KYH, diuretics was used by 3.2% on its own and 22.5% were at least treated with a diuretic. In Tromsø 7, the same proportions were 5.2 and 13.1%. A total of 43 participants (2.7%) in KYH and 14 (0.5%) in Tromsø 7 were treated with ACE and ARB concomitantly. No significant associations were found between uncontrolled hypertension status and being treated with either a monotherapy or combination therapy (Table S1).

Multivariate logistic regression models showed the following factors to be associated with uncontrolled hypertension among those treated for hypertension in both countries: older age, male gender, alcohol use disorder (AUDIT 8+), and not seeing a primary care doctor in the past year (Table 3). Additionally, the absence of CVD history was associated with uncontrolled hypertension in Tromsø 7. No significant interactions were found between gender and age group or age group and CVD history for the two outcomes in either study.

**Table 3:** Logistic regression models of uncontrolled versus controlled hypertension in: Know Your Heart (KYH, Russia, *N* = 1584) relative to Tromsø 7 (T7, Norway, *N* = 2792)

Characteristic	Level	KYH Russia		T7 Norway	
		AOR gender/age	AOR gender/age/CVD	AOR gender/age	AOR gender/age/CVD
Age group	40–49 yr	Ref		Ref	
	50–59 yr	1.25 (0.90;1.74)		1.13 (0.88;1.44)	
	60–69 yr	**1.45** (1.07;1.98)		**1.53** (1.22;1.91)	
Gender	Female	Ref		Ref	
	Male	**1.82** (1.48;2.24)		**1.25** (1.08;1.46)	
CVD history	No	Ref		Ref	
	Yes	0.83 (0.67;1.02)		**0.65** (0.54;0.78)	
Education	Elementary	Ref	Ref	Ref	Ref
	Lower intermediate	0.97 (0.67;1.42)	0.97 (0.67;1.43)	0.92 (0.75;1.12)	0.91 (0.74;1.11)
	Higher intermediate	0.79 (0.58;1.07)	0.79 (0.58;1.07)	0.96 (0.75;1.21)	0.96 (0.76;1.21)
	Graduate	0.79 (0.58;1.08)	0.78 (0.57;1.07)	0.81 (0.65;1.02)	0.81 (0.64;1.00)
Financial constraints	Constrained	Ref	Ref	Ref	Ref
	Intermediary	0.97 (0.75;1.24)	0.95 (0.74;1.23)	N/A	N/A
	Rel. unconstrained	0.96 (0.72;1.29)	0.94 (0.70;1.26)	N/A	N/A
Single	No	Ref	Ref	Ref	Ref
	Yes	0.95 (0.76;1.19)	0.96 (0.77;1.21)	1.06 (0.88;1.27)	1.07 (0.89;1.43)
Smoking	No	Ref	Ref	Ref	Ref
	Yes	0.92 (0.71;1.19)	0.92 (0.71;1.20)	0.81 (0.64;1.02)	0.83 (0.65;1.05)
Alcohol use disorder	Non-drinker past year	Ref	Ref	Ref	Ref
	Low (AUDIT< 8)	1.13 (0.85;1.79)	1.11 (0.84;1.47)	1.16 (0.97;1.39)	1.16 (0.97;1.39)
	High (AUDIT 8+)	**1.71** (1.15;2.54)	**1.69** (1.13;2.51)	**1.35** (1.04;1.74)	**1.37** (1.06;1.78)
Body Mass Index	Under/Normal (< 25)	Ref	Ref	Ref	Ref
	Overweight (25–29)	1.32 (0.97;1.81)	1.32 (0.96;1.80)	1.03 (0.83;1.29)	1.03 (0.83;1.28)
	Obese (30–34)	1.32 (0.96;1.82)	1.32 (0.96;1.82)	1.16 (0.92;1.47)	1.16 (0.92;1.47)
	Very obese (35+)	1.19 (0.83;1.69)	1.19 (0.84;1.71)	1.28 (0.96;1.70)	1.26 (0.95;1.68)
Diabetic	No	Ref	Ref	Ref	Ref
	Yes	0.93 (0.74;1.18)	0.95 (0.75;1.19)	1.03 (0.83;1.27)	1.03 (0.83;1.27)
Seen GP past year	No	Ref	Ref	Ref	Ref
	Yes	**0.58** (0.44;0.75)	**0.59** (0.45;0.76)	**0.67** (0.50;0.90)	**0.67** (0.50;0.91)
CKD	No	Ref	Ref	Ref	Ref
	Yes	1.25 (0.76;2.08)	1.27 (0.77;2.08)	1.09 (0.73;1.65)	1.11 (0.74;1.68)

In KYH, aTRH was associated with older age, CVD history, severe obesity (BMI 35+), diabetes, and CKD (Table 4). In Tromsø 7, aTRH was associated with older age, male gender, CVD history, any drinking, obesity (BMI 30–34) and severe obesity, and diabetes.

**Table 4:** Logistic regression models of aTRH versus non-aTRH in: Know Your Heart (KYH, Russia, *N* = 1584) relative to Tromsø 7 (T7, Norway, *N* = 2792)

Characteristic	Level	KYH Russia		T7 Norway	
		AOR gender/age	AOR gender/age/CVD	AOR gender/age	AOR gender/age/CVD
Age group	40–49 yr	Ref		Ref	
	50–59 yr	1.00 (0.56;1.81)		1.52 (0.84;2.77)	
	60–69 yr	**1.77** (1.04;3.02)		**2.47** (1.43;4.27)	
Gender	Female	Ref		Ref	
	Male	1.12 (0.81;1.53)		**1.41** (1.04;1.91)	
CVD history	No	Ref		Ref	
	Yes	**2.42** (1.74;3.37)		**1.78** (1.29;3.89)	
Education	Elementary	Ref	Ref	Ref	Ref
	Lower intermediate	1.06 (0.59;1.89)	1.03 (0.57;1.85)	0.81 (0.54;1.21)	0.82 (0.55;1.22)
	Higher intermediate	1.09 (0.69;1.74)	1.08 (0.68;1.72)	0.89 (0.57;1.41)	0.89 (0.57;1.41)
	Graduate	0.97 (0.59;1.58)	1.03 (0.63;1.69)	1.17 (0.77;1.76)	1.18 (0.78;1.78)
Financial constraints	Constrained	Ref	Ref	Ref	Ref
	Intermediary	0.99 (0.67;1.47)	1.06 (0.71;1.58)	N/A	N/A
	Rel. unconstrained	1.11 (0.71;1.75)	1.29 (0.82;2.05)	N/A	N/A
Single	No	Ref	Ref	Ref	Ref
	Yes	0.80 (0.56;1.15)	0.77 (0.53;1.11)	1.17 (0.82;1.67)	1.15 (0.80;1.65)
Smoking	No	Ref	Ref	Ref	Ref
	Yes	0.69 (0.43;1.06)	0.68 (0.43;1.06)	0.77 (0.46;1.27)	0.73 (0.44;1.22)
Alcohol use disorder	Non-drinker past year	Ref	Ref	Ref	Ref
	Low (AUDIT< 8)	0.93 (0.59;1.46)	1.02 (0.64;1.60)	**1.44** (1.01;2.05)	**1.45** (1.02;2.07)
	High (AUDIT 8+)	1.00 (0.55;1.81)	1.10 (0.60;2.02)	**1.65** (1.03;2.65)	**1.63** (1.01;2.63)
Body Mass Index	Under/Normal (< 25)	Ref	Ref	Ref	Ref
	Overweight (25–29)	1.28 (0.71;2.30)	1.32 (0.73;2.37)	1.28 (0.76;2.14)	1.28 (0.76;2.16)
	Obese (30–34)	1.73 (0.97;3.10)	1.75 (0.97;3.15)	**1.94** (1.15;3.29)	**1.95** (1.15;3.31)
	Very obese (35+)	**3.15** (1.73;5.73)	**3.10** (1.70;5.68)	**3.84** (2.19;6.72)	**3.97** (2.27;6.97)
Diabetic	No	Ref	Ref	Ref	Ref
	Yes	**2.40** (1.74;3.32)	**2.31** (1.67;3.26)	**2.31** (1.64;3.24)	**2.32** (1.65;3.27)
Seen GP past year	No	Ref	Ref	Ref	Ref
	Yes	1.26 (0.82;1.92)	1.14 (0.74;1.75)	1.43 (0.74;2.75)	1.42 (0.73;2.74)
CKD	No	Ref	Ref	Ref	Ref
	Yes	**2.29** (1.27;4.13)	**2.20** (1.21;4.00)	1.56 (0.81;3.00)	1.52 (0.79;2.93)

## Discussion

The prevalence of hypertension was found to be very high in the Russian population-based study, Know Your Heart (KYH), and this is consistent with other data from Russia [2, 3, 20]. Among those taking antihypertensives in both KYH and Tromsø 7, there were high proportions of individuals with uncontrolled hypertension; although higher in the Russian study (47.8%), compared to the Norwegian study (38.2%). The findings for KYH are consistent with a concurrent cross-sectional study of 25–64 year olds in four other regions of Russia, ESSE-RF-2 Study [3]. This study found that 50.3% of those treated for hypertension were uncontrolled.

Among all those with hypertension, blood pressure control was achieved in 22% of men (KYH and Tromsø 7), while among women it was 33% in Tromsø 7 and 43% in KYH. The concurrent Russian ESSE-RF-2 Study found lower levels of hypertension control but similar to those in KYH, i.e. 16.5% in males and 34.1% in females [3]. In comparison, a study with data from 123 nationally representative surveys of 40–79 year olds in 12 high income countries [21] found that control in males ranged from 17% in Ireland to 69% in Canada. For females, from 26% in Ireland to 58% in Germany. Both KYH and Tromsø 7 would, in other words, be at the bottom of the range, except that females in KYH would be in the middle of this range.

Monotherapies were widely used in both countries, 58% in Tromsø 7 and 44% in KYH. This is contrary to the current European treatment guidelines, which recommend a diuretic to be combined with a drug acting on the renin system (ACE or ARB) or that at least two different drug classes are to be combined [6]. For comparison, the prevalence for monotherapy use in the US (2009–2014) was only 37% [12].

Those with uncontrolled hypertension were less likely to have visited a primary care doctor in the past year than those with controlled hypertension. Poor adherence to antihypertensive therapies may also play a role in the levels of uncontrolled hypertension observed in both studies. Many of the factors found to be associated with uncontrolled hypertension overlap with known factors for poor drug adherence [19, 22] and health check non-attendance [23], i.e. male gender, no primary care visit in the past year, problem drinking, and absence of CVD history. Similarly, poor adherence to lifestyle changes in terms of e.g. weight loss, exercise, dietary salt reduction, etc. could compound the effectiveness of hypertension control for individuals. A recent review of Russian hypertension research found evidence that lower education and single status were associated with lower adherence but differences were not significant in this study [19], perhaps because of insufficient power to detect such a difference.

The prevalence of aTRH was 9.8% in KYH and 5.7% in Tromsø 7. For comparison, the prevalences of aTRH were 14.5% in a US population-based study (2005–2008) [11] and 6.4% in a UK primary care database study [24]. The UK and US studies included those aged above 70 years and may thus be inflated relative to the present study of 40–69 year olds.

The factors associated with aTRH were indicative of patient level factors, i.e. CVD history, obesity, and diabetes. These associations are well-known in the literature, as hypertension is harder to control in patients with obesity and some co-morbidities [6, 11]. Patients with CKD are also known to be at risk of circulatory system conditions including aTRH [11]. The odds ratios for CKD was found statistically significant in KYH, but not in Tromsø 7.

There were some differences in the factors associated with aTRH between the two countries. The associations with CVD history was stronger in Russia than in Norway. This suggests that the Russian participants with aTRH had higher levels of comorbidity. There was only an association of aTRH with alcohol use disorder in Norway. One possible explanation for this difference could be a higher proportion of ‘sick quitters’ in the Russian study population, i.e. individuals who stop a health harming behaviour upon diagnosis with a related health condition.

Interventions to improve hypertension control confront different challenges and opportunities in these two countries. Russia has a general health check programme [5], while Norway does not. In Norway antihypertensive medicines are only available on prescription, whereas in Russia it is possible to obtain any marketable antihypertensive from a pharmacy over-the-counter (OTC). A drug may thus be taken either because it was prescribed or the patient chose, perhaps on the recommendation of a pharmacist, to purchase it [7]. Moreover, patients with hypertension in Norway are reimbursed most of the prescriptions costs [25], while only certain groups including war veterans and recipients of the minimum state pension are reimbursed in Russia [7].

Pharmacists play a particularly important role in the Russian health system, something only a few studies have to date looked at, such as the quality of advice given to patients and opportunities for follow-up.

Antihypertensive combinations that included both ACE inhibitors and ARB were used in both study populations contrary to European treatment guidelines [6]. These two agents both act through the renin-angiotensin system and as a combination not as effective as if patients are treated with drugs with complementary mechanisms. Patients using this combination are also more likely to experience adverse renal events including kidney failure [6]. In absolute terms, this contraindicated co-prescription was relatively rare; 2.7% in the Russian study and 0.5% in the Norwegian one. Electronic prescribing, which includes alerts to potential problems, is used in primary care and pharmacies in Norway, which may explain why this combination was rarer in Norway than Russia. Greater use of multi-drug combinations could potentially reduce prescription errors and improve adherence by reducing the “pill burden” for patients [26], but typically reduces treatment options in terms of dose, formulation, and more individualised prescribing. A recent review of Russian antihypertensive adherence studies however concluded that patient education, telephone reminders, home blood pressure monitoring, and fixed drug combinations were the most important factors for improving adherence [19].

A range of lifestyle factors are associated with hypertension and are assessed as part of standard medical advice, e.g. initiating weight loss, increasing exercise, and reducing dietary salt intake [27, 28]. Adherence to lifestyle change recommendations is thus another potential avenue for future research [29].

Analysis of different therapies by aTRH status was limited in this study by the relatively small sample size and should be replicated in a large database study to properly assess the treatment regimens for this patient group.

### Limitations

Sampling bias introduced by non-response in KYH was assessed by comparing the realised sample against data from the Russian Census 2010 on age, gender, and higher education attainment [8]. Overall, the realised sample for the health check was close to equity, ratio of 0.99 (95% CI 0.93–1.06) for Arkhangelsk and 1.26 (1.17–1.34) for Novosibirsk.

Determination of uncontrolled hypertension and treatment resistance in a survey differs from that in clinical practice, where the treatment regimen, any side effects, and adherence would be reviewed together, perhaps with advice on other actions such as home blood pressure monitoring. This is a limitation that should be borne in mind when interpreting the findings from this and similar studies.

Blood pressure can be spuriously elevated in apprehensive individuals, the so-called white coat effect [30]. This is a type of measurement error is an inevitable limitation of studies such as this where all measurements are done in a clinical setting in a single sitting. To reduce bias from this source, only the mean of the last two of three measurements was analysed.

Antihypertensive use was based in part on self-reported medication data and, as such, potentially prone to recall and other reporting biases. There is however evidence in the literature that self-reports of CVD medication are accurate [31, 32].

## Conclusion

There were high levels of uncontrolled hypertension in both countries, although more so in the Russian than the Norwegian study population. Antihypertensive monotherapies were commonly used in both countries counter to European treatment guidelines, especially in Norway. Our findings suggest considerable scope for promoting the use of combination therapies for those uncontrolled on a single drug. The relatively high proportions of patients not controlled despite being on multiple antihypertensive drugs, furthermore, points to the need to invite patients for individual review of their treatment and any barriers they face adhering to treatment. Further studies should thus include non-adherence in those with uncontrolled hypertension as well as a more in-depth study of patients with aTRH.

## Supplementary information

**Additional file 1: Figure S1**. Sample selection flow diagram. Hypertension was defined as self-reported antihypertensive use or high blood pressure (140+/90+ mmHg). **Table S1.** Age- and gender-adjusted odds ratios (AOR) of uncontrolled versus controlled hypertension and the association with antihypertensive drug class combinations by study: Know Your Heart (KYH, Russia) and Tromsø 7 (T7, Norway).

## Data Availability

The data that support the findings of this study are available from Know Your Heart and The Tromsø Study, but restrictions apply to the availability of these data, which were used under license for the current study, and so are not publicly available. Data from the Know Your Heart Study are however available from the authors upon reasonable request and with permission of Know Your Heart [33]. For The Tromsø Study, data are available subject to scientific and ethical approval of a study protocol [9].

## Abbreviations

ACE: ACE inhibitors ATC code C09A/B
AH: Antihypertensives ATC code C02
ARB: Angiotensin II receptor blockers ATC code C09C/D
ATC: International WHO anatomical therapeutic chemical classification system
aTRH: Apparent treatment resistant hypertension
AUDIT: WHO alcohol use disorders identification test
BB: Beta blockers ATC code C07
CCB: Calcium channel blockers ATC code C08
CKD: Chronic kidney disease
CVD: Cardiovascular disease
DIU: Diuretics ATC code C03
eGFR: Glomerular filtration rate
KYH: Know your heart Study
OTC: Over the counter
