# Supplementary material for: Uncontrolled and apparent treatment resistant hypertension: a cross-sectional study of Russian and Norwegian 40–69 year olds
Source: BMC Cardiovasc Disord. 2020 Mar 13;20:135. doi: 10.1186/s12872-020-01407-2 (PMC7071707; doi:10.1186/s12872-020-01407-2)
Supplement: Supplementary file 1 — Additional file 1: Figure S1. Sample selection flow diagram. Hypertension was defined as self-reported antihypertensive use or high blood pressure (140+/90+ mmHg). Table S1. Age- and gender-adjusted odds ratios (AOR) of uncontrolled versus controlled hypertension and the association with antihypertensive drug class combinations by study: Know Your Heart (KYH, Russia) and Tromsø 7 (T7, Norway). [file 12872_2020_1407_MOESM1_ESM.docx]

**Supplementary materials**


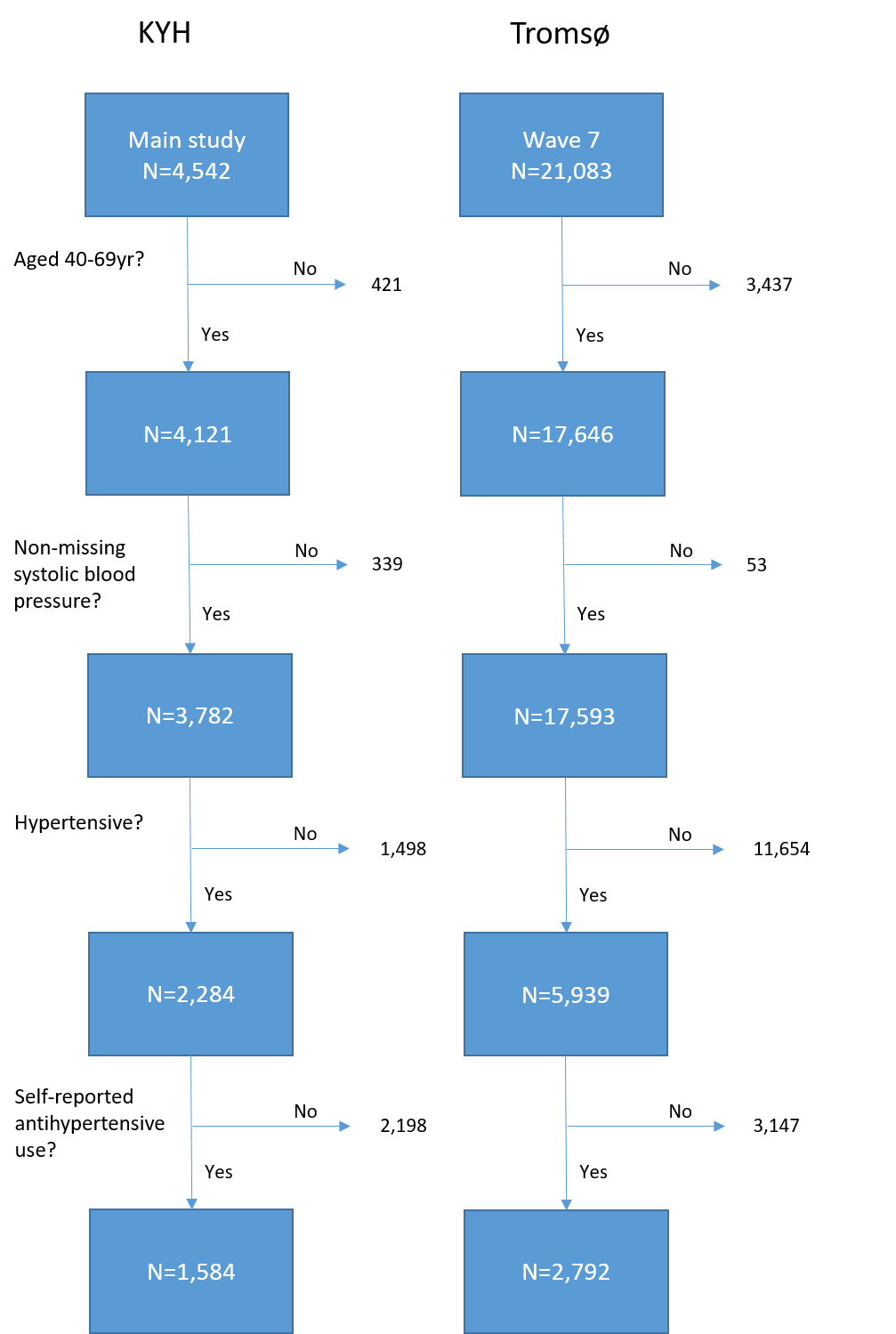


*Figure S1 Sample selection flow diagram. Hypertension was defined as self-reported antihypertensive use or high blood pressure (140+/90+ mmHg).*

Table S1 Age- and gender-adjusted odds ratios (AOR) of uncontrolled versus controlled hypertension and the association with antihypertensive drug class combinations by study: Know Your Heart (KYH, Russia) and Tromsø 7 (T7, Norway).

| **Antihypertensive therapy** | **KYH Russia** |  |  |  |  | **T7 Norway** |  |  |  |  |
| --- | --- | --- | --- | --- | --- | --- | --- | --- | --- | --- |
|  | **Controlled** | **Uncontrolled** | **AOR** | **P-value** | **CI 95%** | **Controlled** | **Uncontrolled** | **AOR** | **P-value** | **CI 95%** |
|  | **N (Col%)** | **N (Col%)** |  |  |  | **N (Col%)** | **N (Col%)** |  |  |  |
| Monotherapies | 355 (44.8) | 339 (42.8) | 0.97 | .745 | (0.79;1.18) | 947 (57.1) | 659 (58.1) | 1.10 | .213 | (0.94;1.29) |
| 2 drug combinations | 278 (35.1) | 301 (38.0) | 1.10 | .362 | (0.90;1.35) | 494 (29.8) | 319 (28.1) | 0.90 | .218 | (0.76;1.06) |
| 3+ drug combinations | 159 (20.1) | 152 (19.2) | 0.91 | .481 | (0.71;1.18) | 217 (13.1) | 156 (13.8) | 0.98 | .881 | (0.79;1.23) |
